# Comparative Pharmacokinetics of Three Bioactive Diterpenoids of *Rabdosia serra* Extract in Normal and Con A-Induced Liver Injury Rats Using UPLC-MS/MS

**DOI:** 10.3389/fphar.2022.944949

**Published:** 2022-07-12

**Authors:** Fangle Liu, Yun Zeng, Pengyu Dai, Kaiwen Huang, Kaihui Zhang, Tao Tao, Meiqi Wang, Chenchen Zhu, Chaozhan Lin

**Affiliations:** ^1^ School of Pharmaceutical Sciences, Guangzhou University of Chinese Medicine, Guangzhou, China; ^2^ School of Basic Medical Sciences, Guangzhou University of Chinese Medicine, Guangzhou, China; ^3^ Guangzhou Chest Hospital, Guangzhou, China

**Keywords:** *Rabdosia serra*, pharmacokinetic differences, diterpenoids, Con A, liver injury, UPLC-MS/MS

## Abstract

*Rabdosia serra* (Maxim.) Hara (*R. serra*), one of the source plants of “Xihuangcao”, has been widely used as a Chinese folk herb with the concomitant function of both medicine and foodstuff for the prevention and treatment of liver disease. Diterpenoids were considered as the major bioactive components in *R. serra*, responsible for their effect on hepatoprotection in previous phytochemical and pharmacological studies, while few comparative pharmacokinetic studies have been conducted under the physiological and pathological conditions. To reveal the difference in the pharmacokinetics process of *R. serra* extract (RSE) in normal and Con A-induced liver injury rats, a rapid ultra-high-pressure liquid chromatography–tandem mass spectrometry method (total running time: 5 min) was established to simultaneously determine three bioactive diterpenoids (enmein, epinodosin, and isodocarpin) in rat plasma. The results showed significant differences in the pharmacokinetic properties of three analytes between the physiological and pathological states. Compared with normal rats, the AUC of the three analytes was remarkably higher in liver injury rats, while the T_max_, T_1/2_, and MRT were shortened. It indicated that RSE has higher exposure and quicker elimination in liver injury rats than that in normal rats. Our results suggested that the pharmacokinetics of hepatoprotective medications was affected by liver injury, which prospected to provide essential information for guiding the healthcare and clinical application of *R. serra* in pathological states.

## 1 Introduction

Liver injury is a common pathological symptom of liver diseases induced by various factors like virals, drugs, and autoimmunity, and its development is often accompanied by inflammation (Qian et al. (2021). Immune liver injury, the main kind of liver injury, is a chronic inflammatory disease that is related to the abnormal immune response of liver cells, which may exist in acute hepatitis that can finally result in liver cancer, liver cirrhosis, and even death (Shojaie et al., 2021). Due to its serious impact on people’s quality of life and the fact that it is difficult for treatment, developing effective medicines for immune liver injury remains a challenge.

Traditional Chinese medicine (TCM) has unique advantages in the clinical treatment of liver injury due to its characteristics of multitarget and multiapproach (Fu et al., 2021). The pharmacokinetic study of TCM is helpful to elucidate the *in vivo* process characteristics of the active ingredients of TCM and clarify the pharmacodynamic material basis of them (Fu et al., 2022). The liver is the crucial detoxifying organ, responsible for the body’s metabolism of foreign substances, including drugs. Therefore, the metabolic process of drugs *in vivo* will be changed, which is affected by the pathological state of liver injury (Alkharfy et al., 2020). The interpretation of integrated intracorporal processes of liver-protective agents in pathological states may be a profound impact on the safety and efficacy of relevant medications in clinical applications.

**FIGURE 1:**
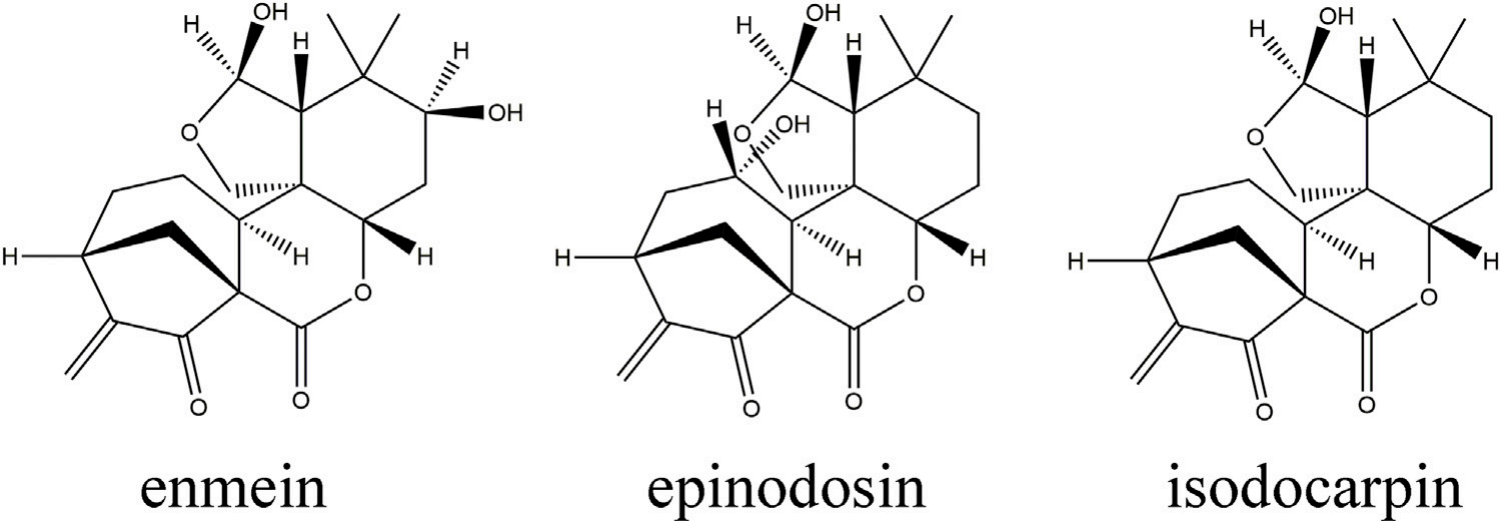
Chemical structures of enmein, epinodosin, and isodocarpin.

*Rabdosia serra*, the dried aerial parts of *Rabdosia serra* (Maxim.) Hara, is one of the sources of “Xihuangcao” and has been commonly used as a medicinal and edible herb for the prevention and treatment of hepatobiliary diseases with the efficiency of heat-clearing, detoxicating, dampness, and moisture reduction for a long time in China (Liu et al., 2020). In clinics, *R. serra* showed a definite curative effect in treating hepatitis, liver injury, intrahepatic cholestasis, and so on (Yang et al., 2021). Modern research studies have also proved that *R. serra* has various pharmacological properties including hepatoprotection, anti-inflammation, antivirus, and immune regulation (Xing et al., 2020; Feng et al., 2021). Among them, liver protection and anti-inflammatory effects of *R. serra* are the most prominent (Sun et al., 2020). Our previous study also showed that *R.* serra extract (RSE) could significantly reduce the levels of AST and ALT and attenuate liver injury induced by concanavalin A (Con A) (He et al., 2016) and alpha-naphthylisothiocyanate (ANIT) (Zhang et al., 2021). However, there is a limited understanding of the comparative pharmacokinetic performance of RSE in physiological and pathological states. Phytochemical studies have presented that terpenoids, phenolic acids, and flavonoids were the predominant composition of *R. serra* (Liu et al., 2020). Diterpenoids, the active and the most characteristic constituents of *R. serra*, have been reported to possess biological effects of hepatoprotective, anti-inflammatory, and antitumor activities, which have attracted great attention from researchers (Wang and Xuan, 2016). Moreover, diterpenoids, such as enmein, isodocarpin, nodosin, epinodosin, and so on, had shown their effects on increasing the cell viability and reducing transaminase content in H_2_O_2_-induced BRL cells, and their strength of efficacy was closely related to their structure according to the 3D-QSAR analysis in our previous research (Liu et al., 2019). Three diterpenoids among them, enmein, epinodosin, and isodocarpin, have a similar structure with the 6,7-seco-ent-kaurene skeleton, and the difference in their structure was in the presence or absence of hydroxyl groups at C-3 and C-11 positions (shown in Figure 1). Thus, the three potential active diterpenoids, enmein, epinodosin, and isodocarpin, were selected as representative components to process the pharmacokinetic study of RSE.

In the present study, we aimed to establish a sensitive and rapid ultra-high-pressure liquid chromatography–tandem mass spectrometry (UPLC-MS/MS) method for the simultaneous determination of three active diterpenoids from RSE in plasma and apply this method for their pharmacokinetic study. According to the comparative pharmacokinetic study of the three bioactive diterpenoids of RSE in normal and Con A-induced liver injury rats, it would be hoped to provide significant information for the application of *R. serra*.

## 2 Materials and Methods

### 2.1 Chemicals and Reagents

The reference standards enmein, epinodosin, and isodocarpin were isolated from *R. serra* and identified by UV, NMR, IR, and MS in our previous research (Lin et al., 2016). The purity of these (>99%) was determined by normalization of their peak areas detected by HPLC. Standard stock solutions of enmein, epinodosin, and isodocarpin at a concentration of 1 mg/ml were diluted with methanol to obtain a reference solution (1 μg/ml) and stored under −20°C until gradient dilution for analysis. Sulfamethoxazole (internal standard, IS) was purchased from Sigma-Aldrich (St. Louis, MO, United States). Concanavalin A (Con A), a reagent for the induction of liver injury in animals, was obtained from Sigma-Aldrich (St. Louis, MO, United States). The dried aerial parts of *R. serra* (voucher specimens NO. IS 20130827) were collected from Guangdong Province and were identified by Professor Chenchen Zhu, a specialist in Pharmacognosy, who worked at Guangzhou University of Chinese Medicine.

MS-grade formic acid, methanol, and acetonitrile were obtained from Merck Co., Ltd. (Darmstadt, Germany). The other chemical reagents (analytical grade) were purchased from Tianjin Zhiyuan Chemical Reagent Co., Ltd. (Tianjin, China). The ultrapure water was prepared using a Milli-Q water purification system (Bedford, MA, United States).

### 2.2 Preparation of *Rabdosia serra* Extract

*R. serra* extract (RSE) was prepared and quality-controlled on the basis of our previous study (Huang et al., 2019). In brief, the herbal samples were crushed and extracted by refluxing with methanol for 2 h. The filtrate was combined and evaporated in vacuum. The concentrated filtrate was partitioned in different solvents; then the crude ethyl acetate (EtOAc) fraction was determined using a Sephadex LH-20 and silica gel column chromatography. The fractions containing diterpenoid components were gathered and collected as RSE used in this study. The contents of enmein, epinodosin, and isodocarpin in RSE extract were analyzed by HPLC at the concentrations of 23.95 , 29.94 , and 112.61 mg/g, respectively (shown in Supplementary Figure S1). The dose of RSE was determined based on the dosage conversion coefficient of rats according to our previous study (Zhang et al., 2021), and a dose of 300 mg/kg was applied in this study.

### 2.3 Animals

Sixteen male Sprague–Dawley (SD) rats (220 ± 20 g) were purchased from the Experimental Animal Center in Guangzhou University of Chinese Medicine [Guangdong, China; license: SYXK (Yue) 2018-0034] and housed in a stable environment (a temperature of 20–24°C, a relative humidity of 50–60%) with a 12 h light–dark cycle. Animal experiments were approved by the Animal Ethics Committee of Guangzhou University of Chinese Medicine (No. 20200201003).

### 2.4 Pretreatment of Plasma

The plasma sample (100 μl) was mixed with 10 μl of IS (300 ng/ml) and 1 ml of ethyl acetate and then vortexed for 3 min. After centrifugation at 12,000 r/min (4°C) for 15 min, the supernatant was dried under the gentle steam of N_2_, and then the residue was dissolved with 100 μl of methanol. Two milliliters of the supernatant were injected into UPLC-MS/MS for analysis.

### 2.5 Instrumentation and Analytical Conditions

An Agilent 6460 triple quadrupole mass spectrometer (Agilent Technologies, Inc., United States) coupled with an electrospray ionization (ESI) source and an Agilent 1260 Infinity UHPLC system (Agilent Technologies, Inc., United States) was used for sample analysis. A Poroshell 120 EC-C_18_ column (3.0 mm × 50 mm, 2.7 μm) was used for sample separation. The mobile phase consisted of acetonitrile (A) and 0.1% formic acid (B) with the gradient elution program as 20% A–60% A (0–5 min) at a flow rate of 0.4 ml/min. The injection volume was 2 μl, and the column temperature was maintained at 25°C.

The positive ionization mode (ESI+) with multiple reaction monitoring (MRM) of the mass spectrometry detector was used, and the parameters were set as follows: drying gas (N_2_) flow rate, 5.1 L/min; drying gas temperature, 300°C; nebulizer pressure, 45.0 psi; capillary voltage, 3370 V; sheath gas temperature, 300°C; sheath gas flow rate, 11.0 ml/min; and nozzle voltage, 0 V.

### 2.6 Quantitative Method Validation

The quantitative method validation, including specificity, linearity, sensitivity, precision, accuracy, recovery, matrix effect, and stability, was performed according to the Food and Drug Administration of the United States (USFDA) guidelines for the validation of a bioanalytical method. Comparison of the chromatograms of standard-spiked samples (three analytes and IS) with and without blank plasma from six different rats was performed to evaluate the specificity. The calibration curves for enmein, epinodosin, and isodocarpin were established by plotting the ratio of the peak area of each compound to IS (Y-axis) versus the concentrations of the compound (eight concentration levels) in the blank plasma sample (X-axis) using the least square linear regression (weighting factor 1/X^2^). The lower limit of quantification (LLOQ) was defined as the lowest concentration with a signal-to-noise ratio (S/N) of 10:1, and a coefficient of variation of no more than 20% should be acceptable. The intraday and interday precision and accuracy were calculated with six replicates of QC samples (low, middle, and high concentrations) on a single day and three consecutive days repeatedly, and the results were displayed as RSD and RE. Three concentration levels of QC samples (20, 100, and 500 ng/ml for enmein and isodocarpin; 25, 125, and 625 ng/ml for epinodosin) and LLOQ samples used in this part were prepared by spiking standard substances to the blank plasma. The recoveries of the three analytes (at three concentration levels of QC) were calculated by comparing the peak areas acquired from the pre-extraction spiked samples with those obtained from post-extraction spiked samples at the same concentration level. The matrix effect was assessed by comparing the peak areas of the three analytes (at three concentration levels of QC) from post-extraction spiked samples with standard solutions of the corresponding concentrations. Finally, the stability of the three concentration levels of QC samples was evaluated under three storage conditions, including storage at 25°C for 24 h, after three freeze-thaws from −80 to 25°C and at −80°C for 1 month.

### 2.7 Induction of Liver Injury Rats

The rats were grouped randomly and equally into the Con A-induced liver injury group (model group) and normal group. The rats of the model group were injected Con A (12.5 mg/kg) *via* the cauda vein, while the rats of the normal group were injected phosphate-buffered saline (PBS) instead. Before the pharmacokinetic study, the Con A-induced rats were evaluated to the established liver injury state by biochemical indicators such as the content of AST and ALT and histopathological observation of liver tissues. The concentrations of AST and ALT were measured using a commercial kit (Nanjing Jiancheng Biotechnology and Science, Inc., China) according to the manufacturer’s instructions. HE staining was performed to observe the histopathological change of liver tissues used per standard procedure (Liu et al., 2021).

### 2.8 Pharmacokinetic Study

After injection of Con A or PBS for 8 h, all the rats were orally administrated with RSE at a concentration of 300 mg/kg. The blood samples were collected from the post-orbital venous plexus veins of each rat into centrifuge tubes containing heparin at a time of 5, 15, 30, 60, 90, 120, 150, 180, 240, 300, 480, 720, and 1440 min after a single dose of RSE and then were centrifuged at 3,800 r/min for 10 min to obtain plasma. All samples were frozen at −80°C until analysis.

### 2.9 Data Analysis

The pharmacokinetic parameters of enmein, epinodosin, and isodocarpin, including the time to reach the maximum drug concentration (T_max_), the maximum plasma concentration (C_max_), half-life (T_1/2_), the area under the plasma concentration–time curve (AUC), and mean residence time (MRT), were calculated by DAS software 3.2.8 (Chinese Pharmacological Association, Anhui, China), and the noncompartmental analysis model was befitted to describe the pharmacokinetic parameters of these three analytes. Data were displayed as the mean ± standard error (SE). Statistical analyses of the data were performed by *Dunnett*’*s t*-test using SPSS 26.0 (IBM Company, Chicago), and *p* < 0.05 was considered statistically significant.

## 3 Results

### 3.1 Quantitative Method Validation

The results of method validation, including specificity, linearity, precision, accuracy, recovery, and matrix effects, showed that this method met the requirements of biological sample analysis.

#### 3.1.1 Specificity

**FIGURE 2:**
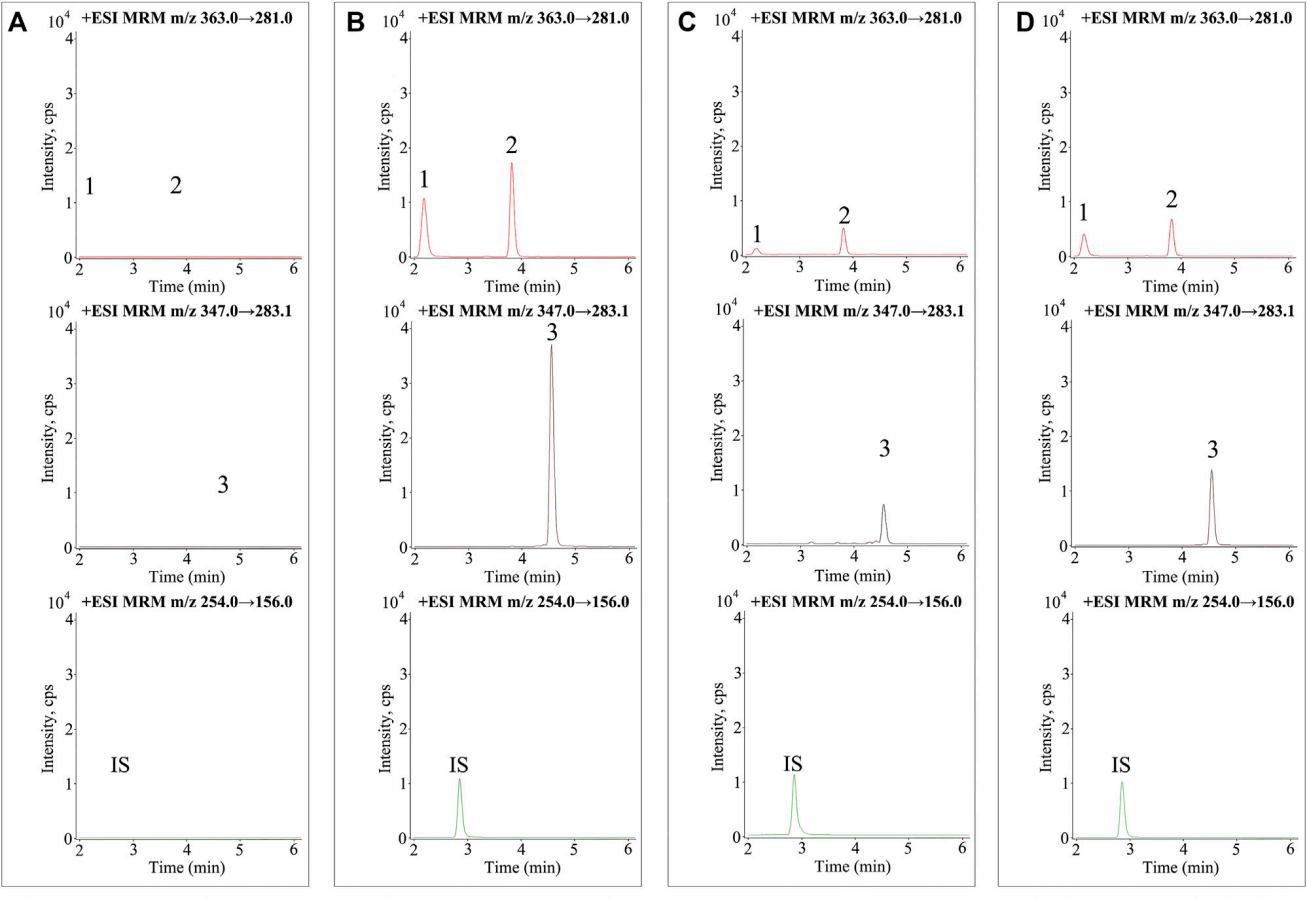
MRM chromatograms of the three analytes and sulfamethoxazole (IS) in **(A)** blank plasma samples, **(B)** blank plasma samples spiked with mixed standards and IS, **(C)** plasma samples obtained from normal rats (2.5 h), and **(D)** plasma samples obtained from liver injury rats (2.5 h). **1**. enmein (RT 2.1 min), **2**. epinodosin (RT 3.8 min), **3**. isodocarpin (RT 4.5 min), and **IS** (RT 2.8 min).

A UPLC-MS/MS method was established for the determination of enmein, epinodosin, and isodocarpin, for which the precursor/product ion pairs of optimized MRM were used as follows: 363.0→281.0 for enmein and epinodosin, 347.0→283.1 for isodocarpin, and 254.0→156.0 for IS (shown in Supplementary Figure S2). The other optimized MRM parameters are listed in Table 1. Representative MRM chromatograms of blank plasma, blank plasma spiked with a mixed standard substance, and plasma from the normal rats and liver injury rats after the oral administration of RSE for 2.5 h are shown in Figure 2, and the retention time (RT) of the three analytes (enmein, epinodosin, and isodocarpin) is 2.1, 3.8, and 4.5 min, respectively. There was no endogenous interference observed in the blank plasma, which exhibited the specificity of the method.

**TABLE 1:** Mass spectrometry parameters of enmein, epinodosin, isodocarpin, and IS.

Analytes	RT (min)	*m/z*	CE	Tube lens
Enmein	2.1	363.0→281.0	17	124
Epinodosin	3.8	363.0→281.0	17	124
Isodocarpin	4.5	347.0→283.1	14	108
IS	2.8	254.0→156.0	27	90

#### 3.1.2 Linearity and Lower Limit of Quantification

As shown in Table 2, the calibration curves presented satisfactory linearity (R^2^ > 0.99) within the concentration range of 2.45–980 ng/ml for enmein, 3.06–980 ng/ml for epinodosin, and 2.65–1060 ng/ml for isodocarpin. The lower limits of quantification (LLOQs) for enmein, epinodosin, and isodocarpin were 2.45, 3.06, and 2.65 ng/ml, respectively, which were appropriate for the quantitation of the three analytes in the pharmacokinetic study.

**TABLE 2:** Linear equation, correlation coefficients (R^2^), linear ranges, and LLOQ of the three analytes.

Analytes	Calibration curve	R^2^	Linear range (ng/ml)	LLOQ (ng/ml)
Enmein	y = 1.9259x + 0.0054	0.9996	2.45∼980	2.45
Epinodosin	y = 2.045x + 0.0087	0.9998	3.06∼980	3.06
Isodocarpin	y = 3.3581x + 0.037	0.9995	2.65∼1060	2.65

#### 3.1.3 Accuracy and Precision

The results of accuracy and precision of the three analytes are displayed in Table 3, which exhibited that the interday and intraday accuracy and precision were less than 13.7% for the three analytes in QC levels. The accuracy and precision of the three analytes were acceptable.

**TABLE 3:** Precision and accuracy of enmein, epinodosin, and isodocarpin in rat plasma (*n* = 6).

Analytes	Concentration added (ng/ml)	Intraday		Interday	
		Precision (RSD, %)	Accuracy (RE, %)	Precision (RSD, %)	Accuracy (RE, %)
Enmein	20	7.6	5.7	13.7	11.0
	100	8.3	7.2	10.2	7.6
	500	5.5	4.2	7.6	5.4
Epinodosin	25	8.3	6.0	10.9	7.6
	125	3.6	3.3	5.4	4.0
	625	3.9	2.9	5.3	3.2
Isodocarpin	20	6.4	4.9	11.6	9.3
	100	5.6	4.6	8.0	8.2
	500	4.4	3.7	8.3	6.9

#### 3.1.4 Recovery and Matrix Effect

As shown in Table 4, the extraction recoveries of enmein, epinodosin, and isodocarpin ranged from 70.1% to 81.8% with RSD less than 10%, while the matrix effects of these were from 100.6% to 103.7% with RSD less than 6%. These results indicated that high extraction recoveries and no obvious matrix effects were observed under the pretreatment method of plasma samples.

**TABLE 4:** Recovery and matrix effect of enmein, epinodosin, and isodocarpin in rat plasma (*n* = 6).

Analytes	Concentration added (ng/ml)	Recovery (%)		Matrix effect	
		Mean ± SD	RSD (%)	Mean ± SD	RSD (%)
Enmein	20	70.1 ± 3.2	5.1	101.0 ± 5.9	5.8
	100	76.3 ± 4.2	6.1	100.6 ± 3.1	3.0
	500	74.8 ± 5.7	9.1	102.2 ± 3.8	3.7
Epinodosin	25	71.3 ± 4.2	6.6	100.6 ± 4.4	4.4
	125	80.6 ± 3.5	4.9	103.4 ± 4.1	4.0
	625	81.2 ± 3.5	4.8	100.7 ± 3.6	3.6
Isodocarpin	20	75.9 ± 4.0	5.8	101.0 ± 3.7	3.6
	100	76.1 ± 4.9	7.2	103.3 ± 5.1	5.0
	500	81.8 ± 4.7	6.4	103.7 ± 4.6	4.5

#### 3.1.5 Stability

The plasma samples containing the three analytes presented good stability (within ±15% variability limits) under different experimental conditions (Table 5).

**TABLE 5:** Stability of enmein, epinodosin, and isodocarpin in rat plasma (*n* = 6).

Compounds (ng/ml)	At 25°C for 24 h		After three freeze-thaw cycles		At −80°C for 30 days	
	Measured conc. (ng/ml)	RSD (%)	Measured conc. (ng/ml)	RSD (%)	Measured conc. (ng/ml)	RSD (%)
Enmein						
20	20.1 ± 0.9	4.4	20.3 ± 1.4	6.8	21.8 ± 1.0	4.6
100	101.4 ± 3.2	3.1	101.5 ± 5.4	5.3	103.1 ± 4.8	4.7
500	503.6 ± 15.2	3.0	501.3 ± 21.4	4.2	510.1 ± 22.1	4.3
Epinodosin						
25	25.7 ± 1.6	6.2	25.3 ± 2.4	9.4	27.0 ± 2.3	8.5
125	125.8 ± 7.4	5.9	126.5 ± 5.4	4.2	128.0 ± 3.2	2.5
625	624.8 ± 23.2	3.7	626.9 ± 25.0	4.0	630.5 ± 35.5	5.6
Isodocarpin						
20	20.5 ± 0.9	4.6	20.5 ± 1.1	5.2	21.3 ± 1.4	6.8
100	102.0 ± 4.5	4.4	102.2 ± 5.1	5.0	105.4 ± 8.7	8.3
500	494.7 ± 23.3	4.7	502.8 ± 11.2	2.2	510.3 ± 30.5	6.0

### 3.2 Model Evaluation

**FIGURE 3:**
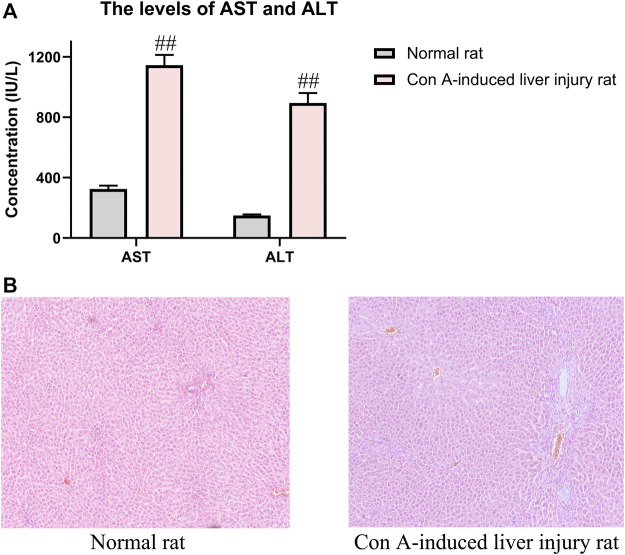
Pharmacological indicators of evaluation of Con A-induced liver injury rats. **(A)** Contents of AST and ALT in normal and liver injury rats. **(B)** Liver histological observation in HE staining of normal and liver injury rats. The data were presented as the mean ± SE (*n* = 8). ##*p* < 0.01 compared with normal rats.

Before the analysis of pharmacokinetics, the Con A-induced liver injury rats were confirmed to be established successfully through the pharmacological parameters such as AST, ALT, and histological observation of liver tissues. As shown in Figure 3, compared with the normal rats, the levels of AST and ALT were significantly increased in the Con A-induced rats. Meanwhile, histological observation of liver tissues indicated that injection of Con A resulted in the development of remarkable liver damage in rats, including hepatocyte necrosis, cellular edema, and inflammatory cell infiltration. These data could prove the successful replication of liver injury rats.

### 3.3 Pharmacokinetic Parameters

**FIGURE 4:**
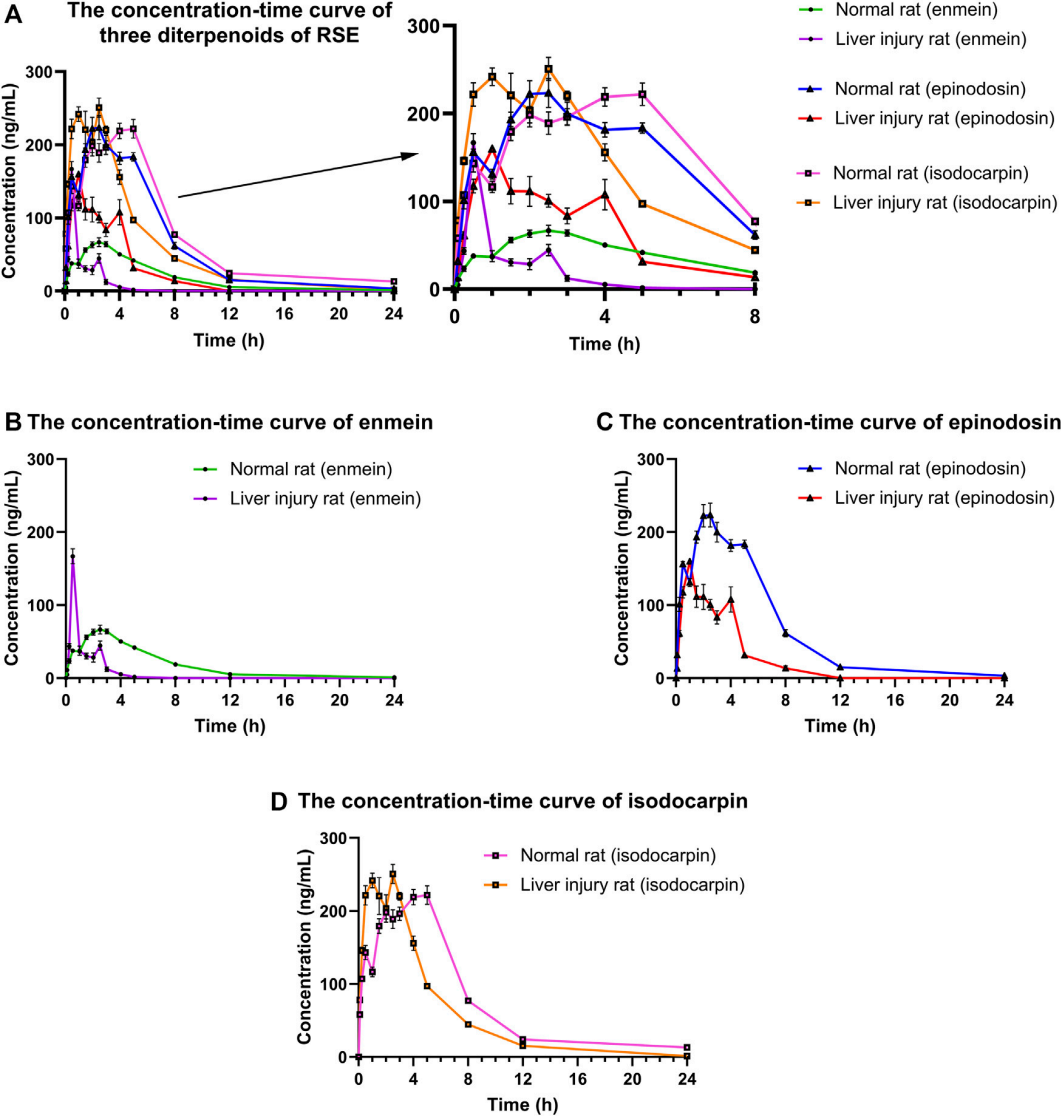
Concentration–time curves of **(A)** three diterpenoids (enmein, epinodosin, and isodocarpin), **(B)** enmein, **(C)** epinodosin, and **(D)** isodocarpin in the plasma of normal and liver injury rats. The data were presented as the mean ± SE (*n* = 8). The rats were given oral administration of RSE (300 mg/kg), and the plasma samples were collected from post-orbital venous plexus veins at 5, 15, 30, 60, 90, 120, 150, 180, 240, 300, 480, 720, and 1440 min after the dose.

The concentrations of enmein, epinodosin, and isodocarpin were determined by using the validated UPLC-MS/MS method in the plasma samples of normal and liver injury rats after the oral administration of RSE and calculated according to the calibration curves. The drug concentration–time curves were drawn and used to display the concentration change profile of the pharmacokinetic process of these three analytes in normal and liver injury rats, which is shown in Figure 4. The pharmacokinetic parameters (Table 6) were calculated by DAS 3.2.8 software using a noncompartmental analysis model according to the concentration–time data.

**TABLE 6:** Pharmacokinetic parameters of enmein, epinodosin, and isodocarpin in the normal rat and liver injury rat (*n* = 8).

Pharmacokinetic parameters	Enmein		Epinodosin		Isodocarpin	
	Normal rat	Liver injury rat	Normal rat	Liver injury rat	Normal rat	Liver injury rat
C_max_ (μg/L)	88.2 ± 35.2	166.78 ± 29.06#	274.9 ± 76.8	182.66 ± 10.02#	267.5 ± 78.1	276.79 ± 34.55
T_max_ (h)	2.5 ± 0.4	0.50 ± 0.13#	2.0 ± 0.5	1.20 ± 0.45	3.9 ± 1.4	2.3 ± 0.45#
T_1/2_ (h)	4.1 ± 1.1	0.87 ± 0.27#	2.3 ± 0.5	1.89 ± 0.21	4.3 ± 1.0	4.06 ± 0.33
MRT_0-**∞**_ (h)	4.9 ± 0.9	1.33 ± 0.43#	4.9 ± 1.0	3.21 ± 0.48#	6.5 ± 1.2	5.01 ± 0.87
AUC_0-t_ (μg/h/L)	432.7 ± 12.9	516.83 ± 24.11#	1635.3 ± 76.6	1781.49 ± 66.69#	1736.0 ± 125.0	2455.95 ± 52.93#
AUC_0-**∞**_ (μg/h/L)	437.2 ± 11.8	597.03 ± 17.28#	1656.8 ± 78.6	1781.59 ± 66.73#	1796.2 ± 182.4	2664.86 ± 65.54#

The data were expressed as mean ± SE. #*p* < 0.05: Compared with normal rats.

After the oral administration of RSE, enmein, epinodosin, and isodocarpin were absorbed slowly and reached C_max_ (88.2 ± 35.2, 274.9 ± 76.8, and 267.5 ± 78.1 μg/L) in about 2.5, 2, and 3.9 h (T_max_) in normal rats, respectively, and then were completely eliminated at approximately 24 h. Moreover, the T_1/2_, MRT_0-∞_, and AUC_0-∞_ of enmein were 4.1 ± 1.1 h, 4.9 ± 0.9 h, and 437.2 ± 11.8 μg/(h·L) in normal rats, respectively; those of epinodosin were 2.3 ± 0.5 h, 4.9 ± 1.0 h, and 1656.8 ± 78.6 μg/(h·L); and those of isodocarpin were 4.3 ± 1.0 h, 6.5 ± 1.2 h, and 1796.2 ± 182.4 μg/(h·L). These results indicated that the three analytes were sufficiently exposed *in vivo* and retained stable retention rather than being rapidly metabolized and eliminated. The C_max_ of enmein, epinodosin, and isodocarpin were 166.78 ± 29.06, 182.66 ± 10.02, and 276.79 ± 34.55 μg/L, respectively, and T_max_ of them were about 0.5, 1.2, and 2.3 h, respectively, in liver injury rats after the oral administration of RSE, independently, which showed that C_max_ of enmein was significantly higher than that in normal rats, while C_max_ of epinodosin and T_max_ of the three compounds were lower than those in normal rats. In addition, the T_1/2_ of these three analytes in liver injury rats were 0.87 ± 0.27 h, 1.89 ± 0.21 h, and 4.06 ± 0.33 h, and MRT_0-∞_ were 1.33 ± 0.43 h, 3.21 ± 0.48 h, and 5.01 ± 0.87 h; they were all obviously shorter than those in normal rats. The AUC_0-∞_ of enmein, epinodosin, and isodocarpin were 597.03 ± 17.28, 1781.59 ± 66.73, and 2664.86 ± 65.54 μg/(h·L), respectively, and these values were higher than those in normal rats. These results suggested that the systemic exposures (C_max_ and AUC) of the three compounds of RSE were increased, and the metabolic and elimination rate (T_max_, T_1/2_, and MRT) were significantly enhanced during the pathological state of liver injury, which might be related to the inflammation and reduction of activity of liver enzymes in liver injury rats.

## 4 Discussion

In this study, a rapid and sensitive UPLC-MS/MS method for the quantification of enmein, epinodosin, and isodocarpin in the plasma samples has been developed and validated, and it was successfully applied to the pharmacokinetic study of these three analytes.

Three potential active diterpenoids, enmein, epinodosin, and isodocarpin, were selected as representative components to process the pharmacokinetic study of RSE. All the three diterpenoids have the 6, 7-seco-ent-kaurene skeleton, and their structures are similar. Enmein and epinodosin are the isomers of each other, and the difference between their structure is that there is a hydroxyl group connected at different positions which are positioned at C-3 (enmein) and C-11 (epinodosin). In addition, isodocarpin lacks this hydroxyl group that causes them to form isomers (Xie et al., 2022). It would be interesting to study the correlation between their pharmacokinetics and their structural characteristics in this research. In addition, a comparative pharmacokinetic study of these three diterpenoids in the normal and liver injury rats after the oral administration of RSE would be helpful in guiding the application of *R. serra*.

According to the plasma concentration–time curves, three diterpenoids showed double or three peaks in both the normal and liver injury rats after the oral administration of RSE. Three reasons may be taken into account in the multipeak phenomenon in the pharmacokinetics of three diterpenoids (Li et al., 2018). First, drugs are absorbed and metabolized by the liver and gastrointestinal tract; they gradually reached the small intestine with gastrointestinal peristalsis, resulting in drug absorption in sequence and leading to the multipeak phenomenon. Second, it might be caused by enterohepatic circulation. Third, this performance of three diterpenoids might be caused by repeated absorption of them due to the biotransformation from other diterpenoids with the same 6, 7-seco-ent-kaurene skeleton existing in RSE after oral administration, which was considered to be the pharmacokinetic characteristic of herbal medicines.

Our previous study reported that the protective effect of diterpenoids from RSE on H_2_O_2_-induced liver injury cells was isodocarpin > epinodosin > enmein (Liu et al., 2019). Pharmacokinetic studies showed that the T_max_, T_1/2_, and MRT of enmein and epinodosin were similar, and those of isodocarpin were longer. Moreover, the C_max_ and AUC of epinodosin and isodocarpin were higher than that of enmein. We thought that it was due to the lipid solubility of epinodosin and isodocarpin, which are linked with or without the hydroxyl group at the C-11 position, and is better than that of enmein, which is linked with the hydroxyl group at the C-3 position, making the former two better to be absorbed into the blood circulation. These results have suggested that the activity of epinodosin and isodocarpin might be superior to that of enmein *in vivo*; it was consistent with our previous results of experiments *in vitro* and the analysis of their 3D quantitative structure–activity relationships (Liu et al., 2019).

Liver injury is a common pathological process that exists in various liver diseases, such as hepatitis, cirrhosis, hepatic fibrosis, trauma, and liver cancer, and it is mainly related to inflammatory response. It is well known that the liver, an important organ for handling the drug metabolism, plays a key role in biotransformation, metabolism, and detoxification of drugs (Marin et al., 2020). The pathological state of liver injury can influence the pharmacokinetic behaviors of drugs (Xia et al., 2021). In this study, the pharmacokinetic characteristics of the three diterpenoids were remarkably different between normal rats and liver injury rats. Based on the pharmacokinetic results, the C_max_ of enmein and isodocarpin in the liver injury rats were higher than that in normal rats after the oral administration of RS, as well as the AUC of the three analytes, which indicated that the systemic exposure of the three analytes was enhanced under the pathological state of liver injury. In addition, the T_max_, T_1/2_, and MRT of the three analytes in liver injury rats were evidently lower than those in normal rats, which suggested that the metabolic rate and elimination rate of these compounds in the model rats might be slowed down. It has been reported that liver injury might result in necrosis of liver cells, decreasing CYP450 enzymes activity (Coutant and Hall, 2018), reducing the first-pass effect (Li et al., 2018), disfunction of drug transporters (Jarvinen et al., 2021), increasing intestinal permeability (Lei et al., 2022), and so on, and then alter the fate of drugs in the body. In particular, about 70–80% of drugs are metabolized through CYP450 enzymes. The abovementioned pathological changes of liver injury might be responsible for the increased exposure of the three compounds in model rats. In addition, a lot of drug transporters and metabolic enzymes are distributed in the liver and participated in the disposition and elimination of drugs. The complex changes (upregulation or downregulation) of these transporters and metabolic enzymes in a pathological state of liver injury may affect the metabolic rate of the drug. For example, upregulation of UDP-glucuronosyltransferases (UGTs) and P-glycoprotein (P-gp) could increase the metabolic rate (Tian et al., 2019), which might be one of the reasons for shortening the parameters of T_max_, T_1/2_, and MRT of the three diterpenoids in liver injury rats. These pathological changes might synthetically result in differences in pharmacokinetic characteristics between the normal and liver injury rats after the oral administration of RSE. Nevertheless, the specific mechanisms are still needed to further investigate and validate.

The development of pharmacokinetic research is conducive to providing a scientific and rational basis for the formulation of drug regimens, and it is also conducive to making more reasonable and accurate predictions of drug toxicity. When formulating a clinical dosing plan, it is necessary to determine the size of the dose and the interval time based on the pharmacokinetics to prevent problems such as drug poisoning due to too many doses or insignificant treatment effects due to too few doses. With regard to the interval between doses, if the interval is too short, it will easily cause the problem of drug accumulation in the patient’s body, and if the interval is too long, the drug will not continue to maintain an effective and reasonable blood concentration level in the patient’s body and ultimately affect the treatment effect. For example, our pharmacokinetics study can provide us with many pharmacokinetic parameters in different states, such as C_max_, AUC, MRT, and T_1/2_, and these results indicated that the exposure and elimination rate of enmein, epinodosin, and isodocarpin were increased under liver-injured states. According to the changes of pharmacokinetics characteristics of RSE, including faster elimination and higher exposure in the liver-injured state than in the normal state, we have to consider whether to decrease the dosage and the dosing interval in clinical administration in order to maintain effective concentrations and avoid toxicity due to the drug’s accumulation. In addition, the pharmacodynamic effect of RSE is a reflection of the extent of its intervention on liver injury, so the relationship between pharmacokinetics (PK) and pharmacodynamics (PD) should be further studied by the PK-PD model, a mathematical tool for constructing *in vivo* concentration–pharmacodynamics correlations, which could provide insight into the PK-PD relationship of RSE and thus better support to protocol design and dosing decisions when RSE or similar herbal drugs are used clinically for the treatment of liver diseases.

The comparative descriptions of pharmacokinetic characteristics of enmein, epinodosin, and isodocarpin in the normal and liver injury rats were helpful to clarify the intracorporal process of RSE and significant for guiding the clinical and healthcare application of *R. serra*.

## 5 Conclusion

To sum up, a sensitive and rapid method for the simultaneous determination of the three active diterpenoids from RSE in plasma samples was developed with method validation and was applied to their pharmacokinetic study. The distinction of pharmacokinetic behaviors of the representative active ingredients of RSE between the physiological state and pathological state was clarified. Compared with the normal rats, the higher exposure and the rapid rate of elimination of the three compounds of RSE in liver injury rats might be due to the pathologic changes. Our results provide significant information on the pharmacokinetic characteristics of RSE and are expected to be useful for the application of *R. serra*.

## Data Availability

The original contributions presented in the study are included in the article/Supplementary Material. Further inquiries can be directed to the corresponding authors.
