# Supplementary material for: Comparative Pharmacokinetics of Three Bioactive Diterpenoids of Rabdosia serra Extract in Normal and Con A-Induced Liver Injury Rats Using UPLC-MS/MS
Source: Front Pharmacol. 2022 Jul 12;13:944949. doi: 10.3389/fphar.2022.944949 (PMC9323086; doi:10.3389/fphar.2022.944949)
Supplement: Supplementary file 1 [file DataSheet1.doc]

Supplementary Material

# Quality assessment of *Rabdosia* serra extract (RSE)

*R.* serra extract (RSE) was prepared and quality controlled on the basis of our previous study (Huang et al. 2019). The main contents are as follows：

1.1 Preparation of RSE

*Rabdosia* serra was crushed and extracted by refluxing with methanol for 2 h, and the filtrate was evaporated under reduced pressure to obtain a residue. The residue was suspended in water and partitioned with ethyl acetate. The EtOAc extract was separated by silica gel column chromatograph eluting with CHCl3-MeOH gradient system (50:1 → 30:1→ 20:1→10:1). The fractions of 30:1 and 20:1 was collected and dried to obtain RSE that enriched the diterpenoids.

1.2 HPLC-DAD Instrumentation and Methods of RSE quantify analysis

HPLC analysis was performed using Agilent 1260 liquid chromatography system (Agilent Technologies, Palo Alto, CA, USA) equipped with quaternary pump, online degasser, autosampler, column temperrture controller, and DAD detector coupled with Kromasil C_18_ column (4.6 mm×250 mm, 5 μm) and analytical workstation. Methanol (A) and water (B) were used as mobile phases at the following gradient elution mode: 0-38 min, 40% A; 38-42 min, 40-55% A; 42-63 min, 55-60% A under flow rate of 0.7 mL/min with 10 μL sample injection.

1.3 Preparation of standard Solutions and RSE samples

Standard solution of three analytes (enmein, epinodosin and isodocarpin) were prepared in methanol at the known concentration that 50 μg/mL. The working solution was diluted with methanol before used, and mixed standard for establish calibration curves.

RSE samples were stored at 4℃ before used. RSE (10 mg) was weighted and dissolved in 10 mL methanol to obtain sample stock solution.

1.4 Results of RSE quantitative determination

HPLC method was applied to determine the contents of three diterpenoids in six batch samples of RSE, and the HPLC chromatograms were shown in Figure S1. The results showed the mean contents of 23.95 mg/g, 29.94 mg/g and 112.61 mg/g for enmein, epinodosin and isodocarpin in RSE.


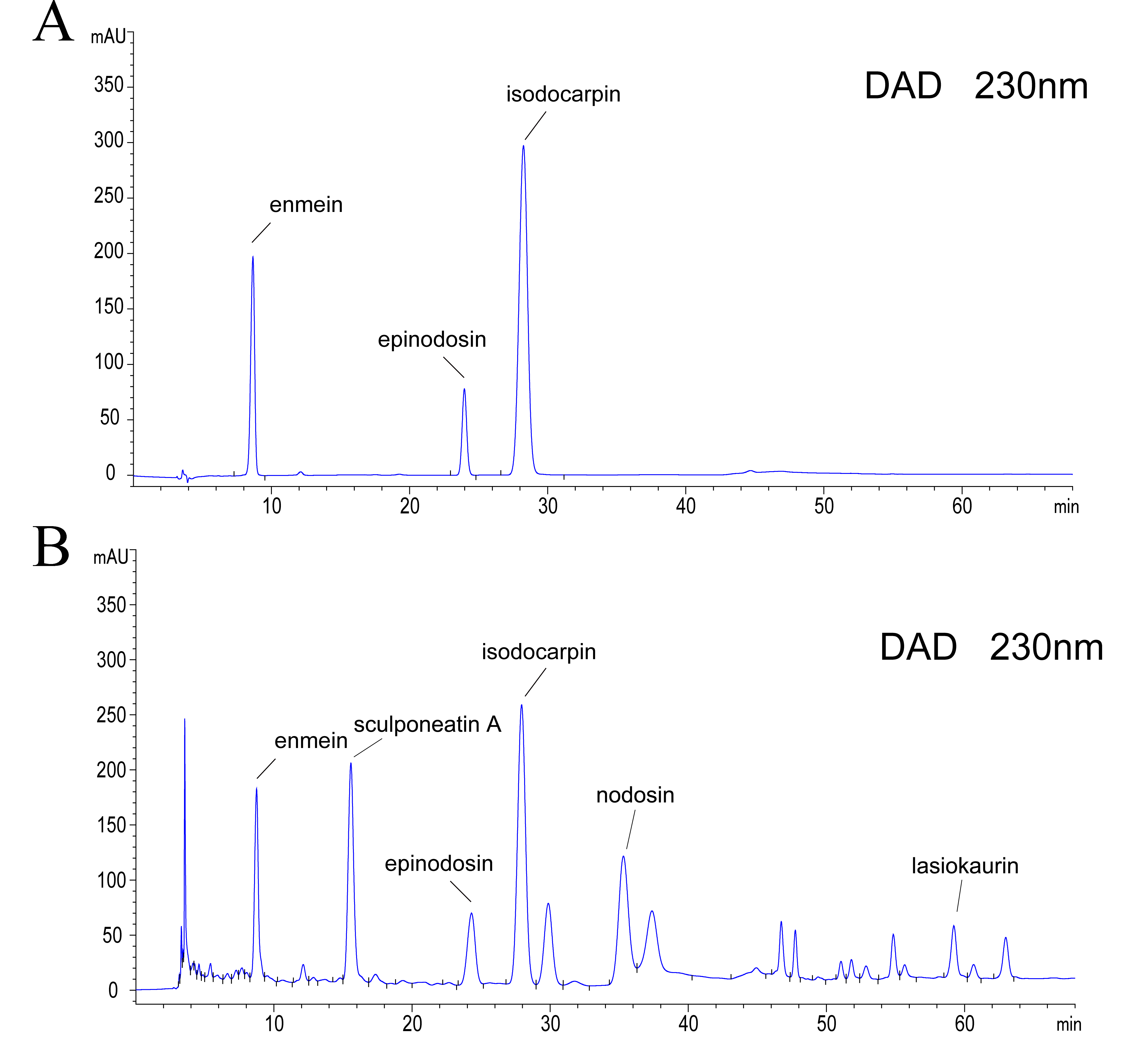


**Supplementary Figure 1 (Figure S1).** HPLC chromatogram of (A) standard substances and (B) RSE sample.

# Optimized MRM

A UPLC-MS/MS method was established for determination of enmein, epinodosin and isodocarpin, which precursor/product ion pairs of optimized MRM were used as follow: 363.0→281.0 for enmein and epinodosin, 347.0→283.1 for isodocarpin, and 254.0→156.0 for IS (shown in Figure S2).


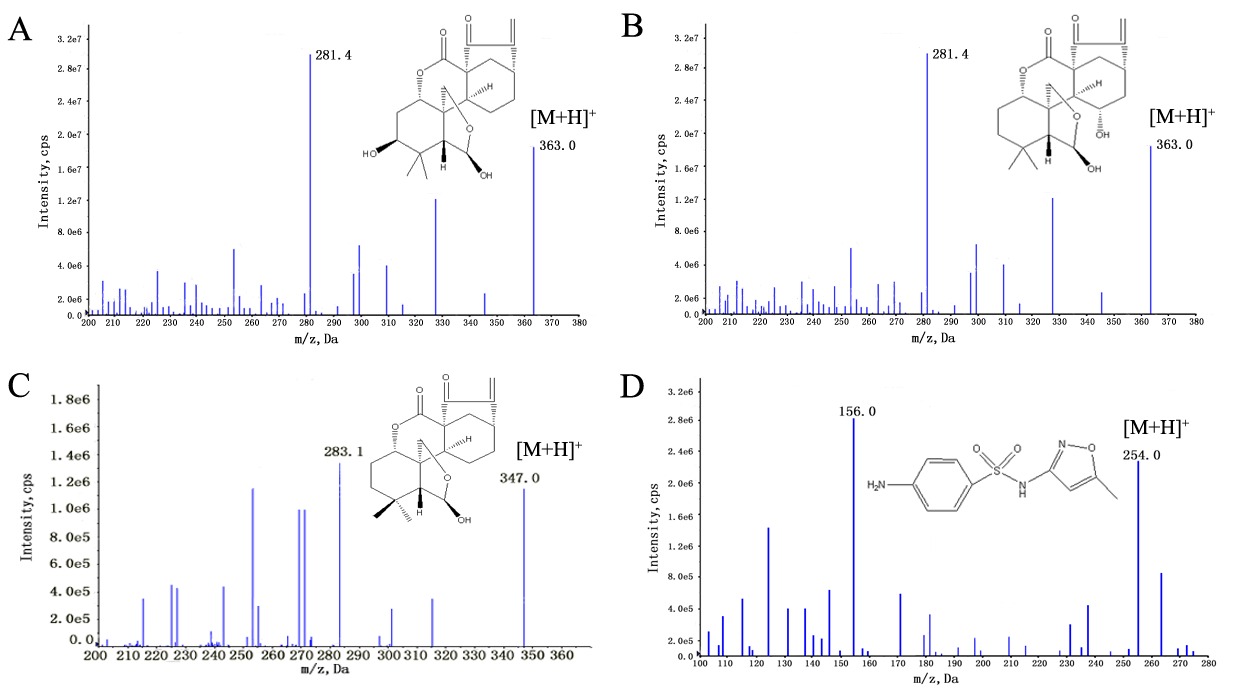


**Supplementary Figure 2 (Figure S2)** The structural formula and the MS/MS spectrometry (A) enmein, (B) epinodosin, (C) isodocarpin and (D) sulfamethoxazole (internal standard, IS).

**Reference**

Huang, T., Wu, C., Chen, D.-J., Lin, C.-Z., and Zhu, C.-C. (2019). Simultaneous Quantitative Determination of Five Diterpenoids Contents in Rabdosia serra by QAMS. *Journal of Chinese Medicinal Materials* 2, 344-347.doi:10.13863/j.issn1001-4454.2019.02.023
